# The preferable shoulder position can isolate supraspinatus activity superior to the classic empty can test: an electromyographic study

**DOI:** 10.1186/s12891-023-06372-3

**Published:** 2023-04-03

**Authors:** Chusak Kijkunasathian, Supajed Niyomkha, Patarawan Woratanarat, Chaiyanun Vijittrakarnrung

**Affiliations:** Department of Orthopedics, Faculty of Medicine Ramathibodi Hospital, Mahidol University, 270, Rama VI Road, Ratchathewi District, Bangkok, 10400 Thailand

**Keywords:** Rotator cuff tear, Supraspinatus tear, Electromyographic Study, Shoulder physical examination, S:D ratio

## Abstract

**Background:**

Supraspinatus (SSP) strength tests are an important shoulder examination tool for clinical evaluations of patients with a suspected SSP tear. While the empty can (EC) test is widely used to diagnose SSP dysfunction, the test cannot selectively activate SSP activity. The aim of this study was to access the electromyographic (EMG) activity within SSP, deltoid, and surrounding periscapular muscles after resisted abduction force to determine which shoulder position helps best isolate SSP from deltoid activity.

**Methods:**

A controlled laboratory EMG study was conducted. Specifically, we conducted an EMG analysis of the seven periscapular muscles (i.e., the middle deltoid, anterior deltoid, SSP, upper trapezius, posterior deltoid, infraspinatus, and pectoralis major) in 21 healthy participants, without any history of shoulder disorder, aged 29 ± 0.9 years old with a dominant-right arm. EMG activities were measured during resisted abduction force according to comprehensive shoulder positions in abduction, horizontal flexion, and humeral rotation. The supraspinatus to middle deltoid (S:D) ratio was calculated using the standardized weighted EMG and the maximum voluntary isometric contraction of the SSP and middle deltoid muscles, for each shoulder position to determine the best isolated SSP muscle strength test position. Results were analyzed with the Kruskal–Wallis test for non-normally distributed data.

**Results:**

Shoulder abduction, horizontal flexion, and humeral rotation significantly affected the activity of the middle deltoid, SSP, and S:D ratio (*P* < 0.05). The S:D ratio increased significantly in lower degrees of shoulder abduction, lower degrees of horizontal flexion, and external humeral rotation over internal rotation. The greatest S:D ratio (3.4 (0.5–9.1)) occurred at the shoulder position of 30° shoulder abduction combined with 30° horizontal flexion and external humeral rotation. Conversely, the classic EC position manifested nearly the smallest S:D ratio (0.8 (0.2–1.2)).

**Conclusion:**

Application of the SSP strength test in the shoulder position of 30 degrees abduction, 30 degrees horizontal flexion, and external humeral rotation offers the best position to isolate the abducting activity of the SSP from that of the deltoid, which could help with diagnosis among patients with chronic shoulder pain with a suspected SSP tear condition.

**Supplementary Information:**

The online version contains supplementary material available at 10.1186/s12891-023-06372-3.

## Background

Rotator cuff tears are a major cause of chronic shoulder disability [1]. Among such tears, the supraspinatus (SSP) is the most affected tendon [2, 3]. An increased recognition and understanding of SSP tear pathology have led to further assessment of the related clinical diagnosis. The SSP muscle strength tests remain an essential tool for the clinical evaluation of patients with a suspected SSP tear. Acting as the main humeral depressor, SSP also functions as a prime initiator of glenohumeral joint elevation [4]. Nevertheless, due to the complex anatomy of the shoulder joint, overlapping muscle function could affect the interpretation of this specific physical examination [5, 6]. Accordingly, physicians must isolate the SSP function from the deltoid abduction force to identify and diagnose SSP tears.

The most common special test used to examine the integrity of SSP is the “empty can” (EC) or “Jobe’s” test. Initially proposed by Jobe and Moynes in 1982 [7], the EC test involves resistance being applied to abduction in 90 degrees shoulder abduction, 30 degrees shoulder horizontal flexion, and full internal humeral rotation. The researchers explained that, with this shoulder position, SSP activity could be isolated based on only one subject [7]. Subsequently, the “full can” (FC) modification test was introduced by Kelly et al. in 1996 [8]. They claimed that FC would be less painful than EC due to avoiding an impingement position, which could result in more reliable results for SSP tear diagnoses.

Traditionally, the shoulder physical examination has been a cornerstone of the diagnostic process. Largely based on the result of these two original studies [7, 8], both EC and FC tests have turned into classic used clinical examination for diagnosing SSP pathology. Nevertheless, the result of their previous EMG studies [7, 8] provided insufficient information to support the conclusion that the EC and FC tests can specifically isolate SSP activity. Certainly, previous EMG studies suggest that the EC and FC tests extremely activate deltoid muscle [9, 10], infraspinatus [9, 11] as well as SSP. In clinical practice, the EC and FC test could be painful and difficult to achieve for patients, resulting in apparent weakness from the pain-mediated reflex inhibitor of the muscle. Many previous studies have manifested the unsatisfactory diagnostic accuracy of these tests in terms of clinical application [5, 12–14). Longo et al. [14] conducted a review article on clinical testing for SSP pathology, they found that the EC test mostly had a sensitivity lesser than 80% (4 out of 6 studies), and a specificity of less than 80% (5 out of 6 studies). Correspondingly, they also found that the FC test mostly had a sensitivity and specificity lesser than 80% (3 out of 4 studies). Some studies also demonstrated sensitivity, specificity, and accuracy of the EC test as low as 30%, 35% and 50%, respectively [8, 15, 16]. In conclusion, the diagnostic accuracy of these classic EC and FC tests are still unsatisfactory, the more proper shoulder position which could isolate and specific with SSP activity should be further determined.

To increase SSP muscle strength test accuracy, the proper shoulder position must be accountable for maximizing the abducting contribution of SSP and minimizing the deltoid abducting activity. Chalmers et al. advocated the SSP and middle deltoid ratio (S:D ratio) as a parameter to represent and quantify how well each shoulder position isolated SSP activity from deltoid activity [17]. Several previous studies showed that a lower degree of shoulder abduction might be more specific to SSP function than to that of the deltoid function [17–19]. However, no previous study examined the potential relationship between periscapular muscles, especially the SSP and deltoid, and comprehensive shoulder position with respect to abduction, horizontal flexion, and humeral rotation. To fill that research gap, the primary objectives of our study were to [1] conduct an electromyographic (EMG) study to determine which shoulder position best isolates SSP from deltoid activity and [2] evaluate the EMG activity within the SSP, deltoid, and surrounding shoulder muscle after resisted abduction force in various shoulder positions. We hypothesized that lower degrees of abduction and horizontal flexion would better isolate SSP abduction activity from the deltoid activity.

## Materials and methods

### Study design and participants

The controlled laboratory EMG study was conducted at the Department of Orthopedics, Faculty of Medicine Ramathibodi Hospital. Participants who were normal healthy individuals aged 18–40 years old without any history of shoulder instability, major shoulder trauma, shoulder surgery, shoulder or periscapular pain were included. A complete shoulder physical examination was performed in every participant by the orthopedic surgeon. Participants with medical comorbidity-affected shoulder motion, or abnormal shoulder examination were excluded. Experimental testing was conducted on a healthy participant as it was determined that interpretation of SSP testing in patients with SSP pathology has to be established base on a precise understanding of normal EMG muscle activation [13]. Besides, our protocol was set up in the same fashion as many previous EMG studies [4, 11, 13, 17].

The sample size was calculated using STATA 15.0 and a reference from a previous study [8]. The following values were used to calculate the sample size: an alpha error of 0.05, power of study of 0.8, mean SSP MVIC in 90° shoulder flexion and external rotation of 7.65 volts, SD of 1.58 volts, mean SSP MVIC in 0° shoulder flexion and external rotation of 6.69 volts, SD of 2.96 volts, 23 measurements, 1 baseline measurement, and a between-measurement correlation of 0.8. The total sample size needed was 21 participants. After informed consent was obtained from patients, baseline characteristics—including age, gender, and body mass index (BMI)—were recorded. A total of 21 participants were included in the final sample. All participants were males aged 29 ± 0.9 years old with a dominant-right arm. The mean BMI was 24.6 ± 2.9 kg/m^2^.

All participants were unaware of the study hypothesis. This study was ethically approved by our hospital’s institutional research board committee (IRB number MURA2017/582). All methods were performed in accordance with the Helsinki guidelines and relevant CIOMS guidelines.

### Experiment protocol

**Fig. 1:**
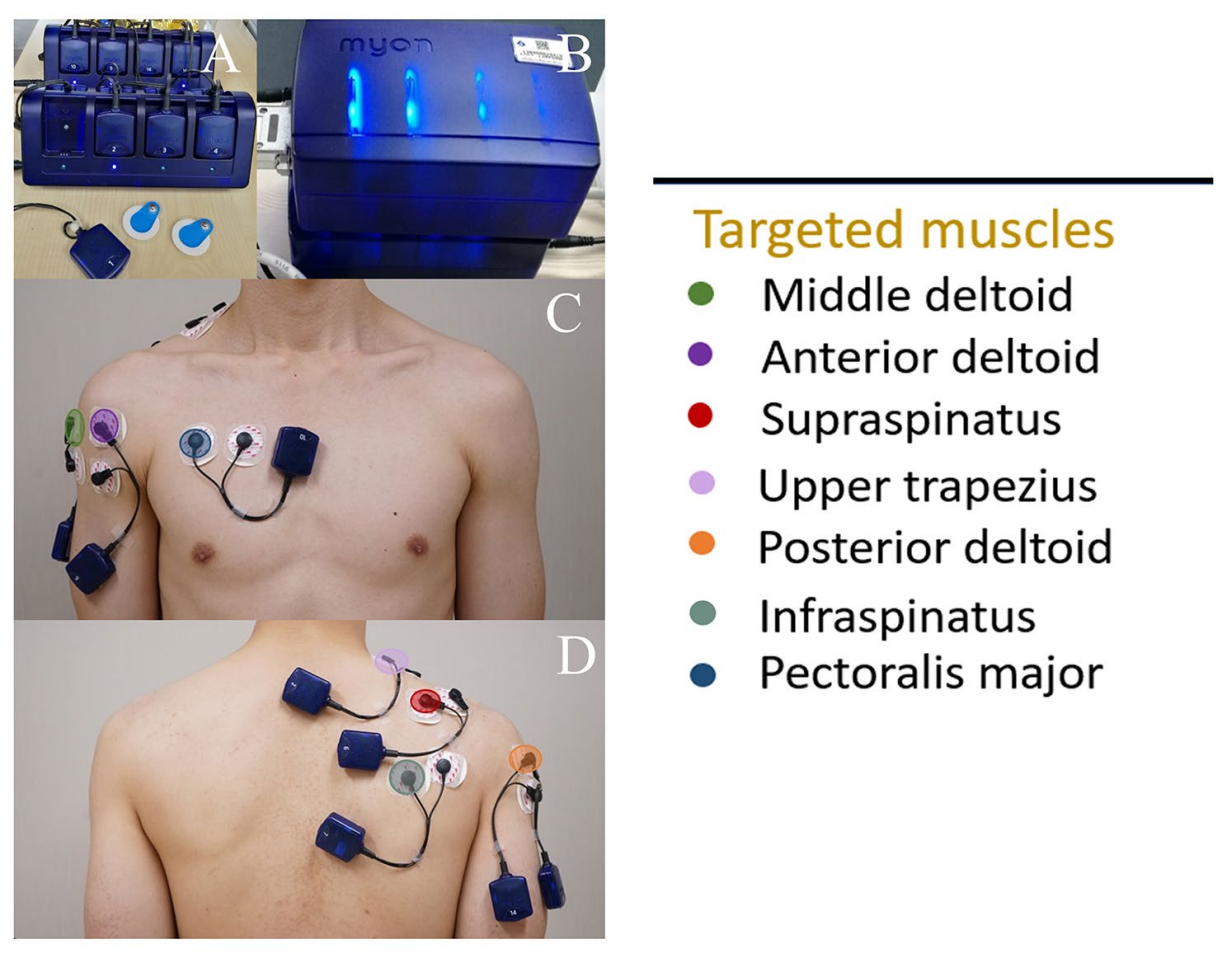
Wireless Myon 320 Surface Electromyography System®; Schwarzenberg, Switzerland. The system consists of transmitters (A) and receivers (B). Surface electrode placement on seven muscles identified by different-colored electrodes for the anterior side (C) and posterior side (D)

All eligible participants dressed in the proper attire and exposed their upper trunks and extremities. Only the dominant arm was considered for measurement. The scapular plane position was measured using a standard goniometer (Supplementary Fig. 1) according to previous proposed method [20]. The skin around the shoulder was prepared using an alcohol rub. After the surface anatomical landmarks were outlined, electromyography (Wireless Myon 320 Surface Electromyography System®; Schwarzenberg, Switzerland) was used; surface-adhesive electrodes (Fig. 1A–B) were applied to skin, parallel to the muscle direction with 2 cm between center-center, as described in a related study [21]. The electrodes were applied over seven muscles (i.e., middle deltoid, anterior deltoid, supraspinatus, upper trapezius, posterior deltoid, infraspinatus, and pectoralis major) by a single experienced physiotherapist (Fig. 1C–D; Table 1). These seven periscapular muscles are selected based on previous relevant EMG studies that demonstrated an activation of these muscles during shoulder abduction [8, 11, 13]. Subscapularis and Latissimus dorsi were excluded due to its prime function as an internal rotation and low activity during shoulder abduction [8, 13, 22]. The EMG signals were sampled by computer at 1000 Hz. Eight integrated channels were used for signal filtering (10 and 400 Hz, Butterworth) and rectification. The isometric contraction was measured for a total of 5 s interval [8].

**Table 1:** Descriptions of the surface electrodes placement and MVIC normalization positions for each of the seven shoulder muscles examined

Muscle	Surface electrode placement [11, 23]	MVIC normalization positions [24, 25]
Middle deltoid	Intersection of the midpoint between the anterior and posterior deltoid muscles and the midpoint between the acromion and deltoid tuberosity	Resisted abduction at 0° elevation in scapular plane and elbow flexion to 90°
Anterior deltoid	3.5 cm below the anterior angle of the acromion	Resisted forward flexion at 0° elevation in scapular plane and elbow flexion to 90°
Supraspinatus	Midpoint and 2 finger-breadths anterior to scapular spine	Resisted elevation at 90° elevation in scapular plane, 45° internal rotation and elbow extension
Upper trapezius	Supero-medial and infero-lateral to a point 2 cm lateral to one-half the distance between the C7 spinous process and the lateral tip of the acromion	Resisted shoulder shrug with subject seated and arm at side
Posterior deltoid	2 cm below the posterior angle of the acromion	Resisted extension at 0° elevation in scapular plane and elbow flexion to 90°
Infraspinatus	Parallel to spine of scapulae, approximately 4 cm below, over the infrascapular fossa	Resisted external rotation at 90° elevation in scapular plane and neutral rotation
Pectoralis major	3.5 cm medial to the anterior axillary line	Resisted horizontal adduction at 90° elevation in scapular plane and elbow flexion to 90°

**Fig. 2:**
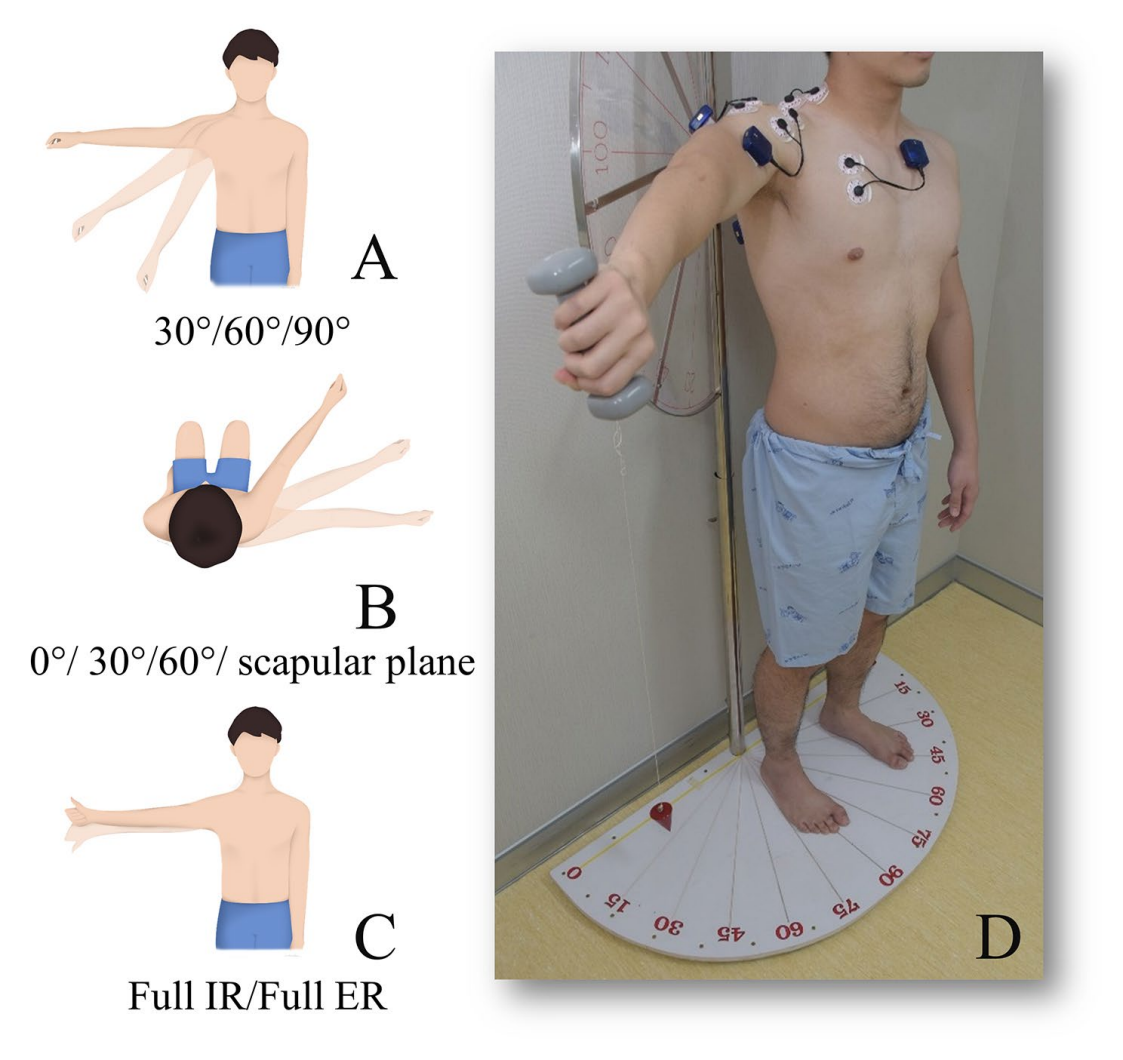
Comprehensive shoulder motion tasks for 30°/60°/90° shoulder abduction (A), 0°/30°/60°/scapular plane horizontal flexion (B), and full internal/external humeral rotation (C). Example of two-plane goniometer measurement of 90° shoulder abduction, 30° horizontal flexion, and full internal humeral rotation, i.e., EC position (D)

A single well-trained examiner conducted all testing to ensure the replication of the same resistance and positions. The maximum voluntary isometric contraction (MVIC) of the seven muscles was measured by performing manual maximum isometric resistance in standard posture references, as described in Table 1. The test order for individual participants was a randomized sequence. Each contraction was set in the same 5-second pattern used in a previous study [11]. Three trials of each muscle testing were conducted, with a minimum rest interval of 30 s between trials. Every trial was closely monitored to avoid compensatory movement from the trunk and scapular. After recording the MVIC value for each muscle reference, examiner instructed participants to isometrically hold a standard 1-kg dumbbell for 5 s in 24 total shoulder positions (i.e., factorial of 30°/60°/90° shoulder abduction; 0°/30°/60°/scapular plane; and internal/external humeral rotation) (Fig. 2A–C). The EMG activity of each muscle after participants held the 1-kg weight was collected as the standardized weighted EMG according to the individual position. The participants’ shoulder angle measurements were done using 2-plane goniometer (Fig. 2D), which has been previously demonstrated excellent reliability and validity when compared with a digital inclinometer for measuring shoulder range of motion [26]. To prevent the fatigue effect, participants were given a minimum 30-second rest interval after each measurement [27].

### Data collection & outcome measurement

We used the EMG activity of the middle deltoid to represent the deltoid abduction activity. The best shoulder position to isolate SSP from deltoid activity was quantified using S:D ratio [17], which can be calculated using a percentage of the standardized weighted EMG (%sEMG) of the SSP divided by the %sEMG of the middle deltoid. The %sEMG of each muscle was calculated using the relevant standardized weighted EMG divided by the relevant MVIC. Thus, the higher S:D ratio represents a higher contribution of SSP activity compared to that of the middle deltoid.

### Statistical analysis

Statistical analyses were calculated using Stata 15 software (StataCorp, College Station, TX, USA). The baseline characteristics—such as age (years), body weight (kg), height (cm), BMI (kg/m^2^), scapular plane (degree), MVIC, and %sEMG—of seven muscles were reported using the mean and standard deviation. Data were analyzed for completeness and normality using the Shapiro–Wilk test combined with a normal distribution plot. The Mann–Whitney test was used for continuous variable that did not satisfy normality. The between-group difference in the MVIC and S:D ratio was determined by analyzing the variable (normally distributed: one-way analysis of variance; non-normally distributed: Kruskal–Wallis test). The multilevel mixed-effects linear regression analysis was used to identify the coefficient comparing groups of higher degrees of motion. Multiple regression with a parsimonious model and 95% confidence interval was used to identify and control confounding factors. A *P* value less than 0.05 was considered statistically significant.

## Results

### Participant demographic data

The total number of recruited samples was 21 participants. The mean scapular angle was 26.2° ± 4.2°. The mean MVIC was 2.2 ± 0.3 volts for SSP and 2.2 ± 0.2 volts for the middle deltoid, respectively. The participant’s demographic data and MVIC values for other muscles are reported in Table 2.

**Table 2:** Participants’ demographic characteristics and the maximum voluntary isometric contraction for each of the seven muscles

Demographic characteristics	Study population
	N = 21
**Age (years)** ^∞^	
Mean ± SD	29.0 ± 0.9
**Gender** ^**µ**^	
Male	21(100%)
**Height (cm)** ^**∞**^	
Mean ± SD	175.6 ± 5.3
**Weight (kg)** ^**∞**^	
Mean ± SD	75.9 ± 10.3
**BMI (kg/m2)** ^**∞**^	
Mean ± SD	24.6 ± 2.9
**Scapular Plane (degrees)** ^**∞**^	
Mean ± SD	6.2 ± 4.2
**Maximum voluntary contraction (unit)** ^**∞**^	
Middle deltoid	2.2 ± 0.2
Anterior deltoid	2.1 ± 0.2
Supraspinatus	2.2 ± 0.3
Upper trapezius	1.4 ± 0.5
Posterior deltoid	2.1 ± 0.4
Infraspinatus	1.6 ± 0.4
Pectoralis major	1.4 ± 0.5

∞: value presented as mean ± standard deviation

µ: value presented as the number of volunteers with that condition (percentage)

### Standardized weighted EMG testing

We normalized all standardized weighted EMG activities to %sEMG, which can be calculated by dividing the sEMG of the activities by their MVIC. The highest percentage %sEMG for SSP was detected in the 90° shoulder abduction combined with 0° horizontal flexion and internal humeral rotation. The %sEMG values for the seven muscles in all shoulder positions are represented in Table 3.

**Table 3:** The percentage of standardized weighted EMG (%sEMG) within all seven muscles (i.e., middle deltoid, anterior deltoid, supraspinatus, upper trapezius, posterior deltoid, infraspinatus, and pectoralis major) regarding comprehensive shoulder positions

Position no.	Abduction (degrees)	Horizontal flexion (degrees)	Rotation	Middle deltoid	Anterior deltoid	Supraspinatus	Upper Trapezius	Posterior deltoid	Infra Spinatus	Pectoralis major
**1**	**30**	**0**	**IR**	**15.0 (7.5)**	**10.7 (8.9)**	**11.3 (4.5)**	**6.7 (5.3)**	**9.7 (4.0)**	**4.8 (7.2)**	**2.7 (1.7)**
**2**	**30**	**0**	**ER**	**7.8 (4.3)**	**6.7 (5.5)**	**11.7 (5.2)**	**8.4 (6.4)**	**4.2 (2.5)**	**4.7 (2.7)**	**2.5 (1.5)**
**3**	**30**	**30**	**IR**	**9.2 (4.6)**	**7.9 (7.9)**	**9.5 (4.1)**	**8.9 (7.4)**	**5.5 (2.6)**	**2.7 (1.3)**	**2.7 (1.8)**
**4**	**30**	**30**	**ER**	**3.6 (2.6)**	**6.8 (4.0)**	**9.3 (4.3)**	**9.2 (8.0)**	**2.2 (3.0)**	**7.3 (11.9)**	**2.7 (1.6)**
**5**	**30**	**60**	**IR**	**8.3 (7.7)**	**6.9 (5.6)**	**10.2 (9.3)**	**14.7 (29.6)**	**3.5 (3.5)**	**4.4 (8.3)**	**3.0 (1.8)**
**6**	**30**	**60**	**ER**	**2.0 (1.1)**	**6.4 (3.6)**	**7.2 (6.4)**	**7.5 (8.2)**	**1.0 (0.5)**	**4.4 (2.8)**	**3.2 (2.0)**
**7**	**30**	**SP**	**IR**	**11.2 (5.1)**	**9.1 (7.4)**	**10.4 (5.0)**	**10.8 (11.6)**	**6.4 (2.5)**	**2.8 (1.1)**	**2.7 (1.7)**
**8**	**30**	**SP**	**ER**	**3.9 (2.1)**	**7.6 (4.5)**	**9.7 (4.8)**	**9.4 (8.4)**	**1.8 (1.2)**	**5.1 (2.8)**	**2.7 (1.5)**
**9**	**60**	**0**	**IR**	**29.7 (14.0)**	**22.5 (16.3)**	**20.1 (8.3)**	**14.0 (9.3)**	**19.2 (7.1)**	**5.9 (2.4)**	**2.8 (1.8)**
**10**	**60**	**0**	**ER**	**12.7 (5.7)**	**12.7 (8.1)**	**17.0 (7.1)**	**16.0(11.2)**	**5.9 (3.8)**	**7.2 (5.9)**	**2.7 (1.6)**
**11**	**60**	**30**	**IR**	**18.4 (6.2)**	**16.3 (12.8)**	**16.9 (6.7)**	**15.4 (10.6)**	**10.9 (4.0)**	**5.4 (2.7)**	**3.8 (4.6)**
**12**	**60**	**30**	**ER**	**9.2 (2.7)**	**12.7 (5.2)**	**14.5 (4.7)**	**17.3(13.6)**	**4.4 (6.5)**	**8.4 (6.0)**	**4.3 (7.1)**
**13**	**60**	**60**	**IR**	**13.2 (7.6)**	**14.2 (11.3)**	**14.8 (8.2)**	**13.8 (11.3)**	**7.4 (3.6)**	**9.2 (7.8)**	**6.7 (8.5)**
**14**	**60**	**60**	**ER**	**8.8 (3.3)**	**14.9 (5.4)**	**12.3 (6.5)**	**15.6(11.0)**	**2.9 (1.7)**	**7.3 (2.8)**	**4.3 (2.4)**
**15**	**60**	**SP**	**IR**	**21.5 (9.8)**	**18.2 (12.6)**	**17.6 (7.1)**	**14.7 (9.6)**	**13.1 (6.2)**	**8.2 (11.0)**	**2.9 (1.7)**
**16**	**60**	**SP**	**ER**	**9.8 (3.3)**	**13.1(5.7)**	**15.6 (6.2)**	**16.6(10.8)**	**3.6 (1.7)**	**7.8 (5.1)**	**2.9 (1.6)**
**17**	**90**	**0**	**IR**	**39.9 (13.4)**	**30.9 (17.6)**	**27.1 (11.9)**	**20.9 (13.8)**	**24.8 (10.1)**	**8.3 (4.0)**	**2.9 (1.6)**
**18**	**90**	**0**	**ER**	**17.2 (8.1)**	**18.3(12.5)**	**22.1 (9.9)**	**20.4(13.7)**	**8.2 (5.6)**	**7.6 (4.5)**	**3.3 (3.2)**
**19**	**90**	**30**	**IR**	**31.7 (11.3)**	**25.6 (17.4)**	**22.7 (9.5)**	**21.4 (13.5)**	**18.8 (8.2)**	**7.9 (3.2)**	**3.0 (1.7)**
**20**	**90**	**30**	**ER**	**15.1 (5.9)**	**17.5 (9.5)**	**18.6 (7.5)**	**22.1(14.7)**	**5.3 (1.7)**	**8.5 (3.9)**	**3.3 (2.1)**
**21**	**90**	**60**	**IR**	**23.2 (8.8)**	**20.9 (11.1)**	**20.3 (10.3)**	**22.3 (14.7)**	**11.8 (4.4)**	**9.6 (6.0)**	**3.2 (1.8)**
**22**	**90**	**60**	**ER**	**14.8 (4.8)**	**19.9 (7.8)**	**15.6 (6.5)**	**23.7(20.0)**	**4.7 (1.6)**	**10.7 (8.7)**	**4.3 (3.2)**
**23**	**90**	**SP**	**IR**	**32.1 (13.0)**	**26.1 (17.6)**	**24.3 (11.8)**	**21.6 (14.7)**	**18.9 (9.2)**	**8.4 (4.1)**	**3.0 (1.7)**
**24**	**90**	**SP**	**ER**	**15.7 (7.1)**	**17.5 (8.0)**	**19.7(10.1)**	**21.2(13.3)**	**5.9 (2.4)**	**8.5 (4.4)**	**3.0 (1.6)**

**Value presented as mean(standard deviation)**

*IR = internal rotation, ER = external rotation, SP = scapular plane

According to the relationship between shoulder positions and %sEMG (Supplementary Fig. 2), the infraspinatus and pectoralis major demonstrated unrelate activity in any shoulder position, illustrated as a nearly flat graph over all positions. Additionally, the upper trapezius and posterior deltoid manifested similar trend to SSP and middle deltoid respectively, but with a relative lower magnitude of EMG changes.

**Fig. 3:**
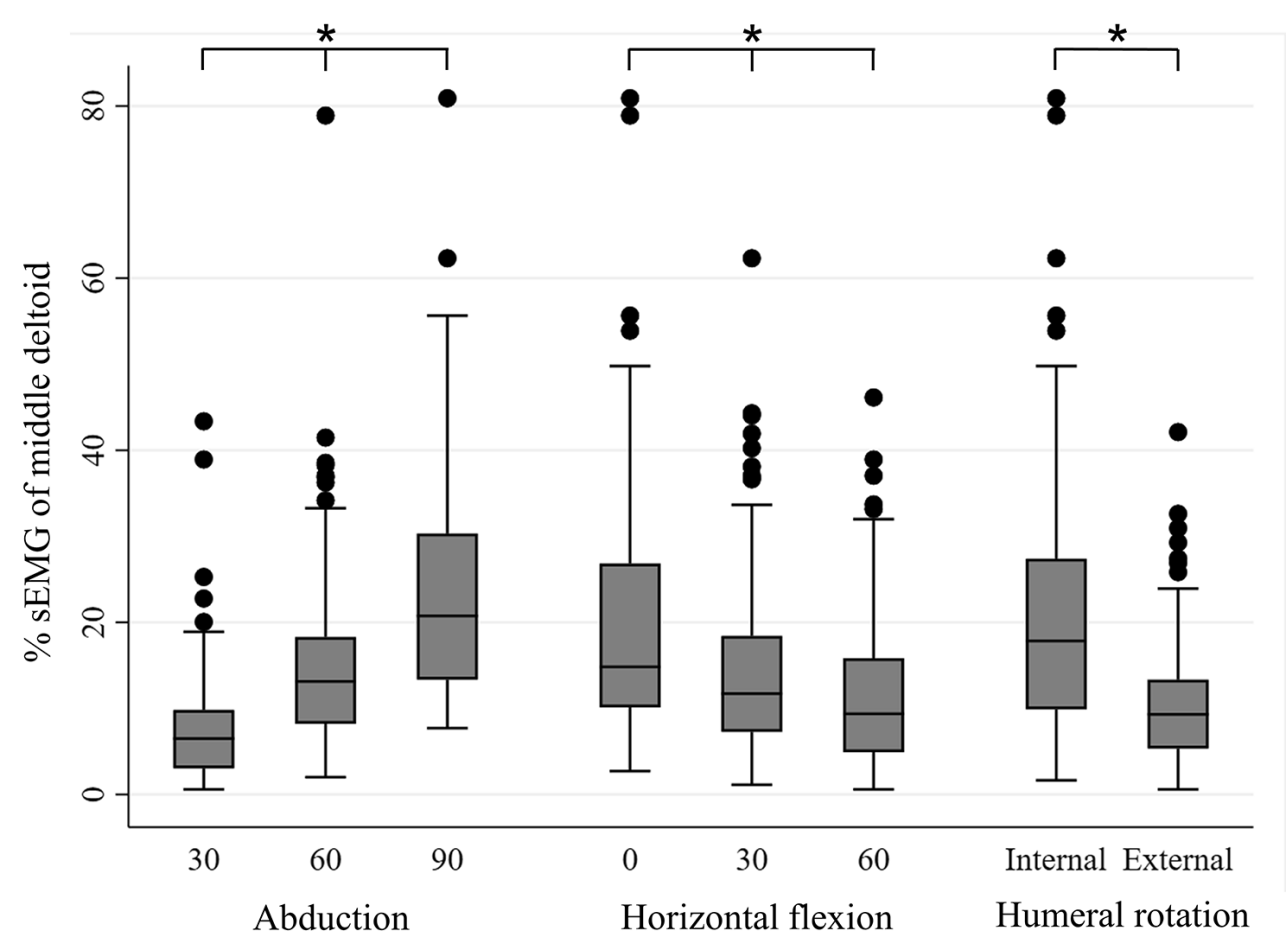
Comparison of the percentage of standardized weighted EMG (%sEMG) of middle deltoid between the groups graded by degree of shoulder abduction, degree of shoulder horizontal flexion, and humeral rotation. Error bars indicate the interquartile range (IQR) of the median. Black dots indicate values above the upper fence (1.5*IQR). * above the lines spanning between groups indicates significant *P-*values < 0.05

**Fig. 4:**
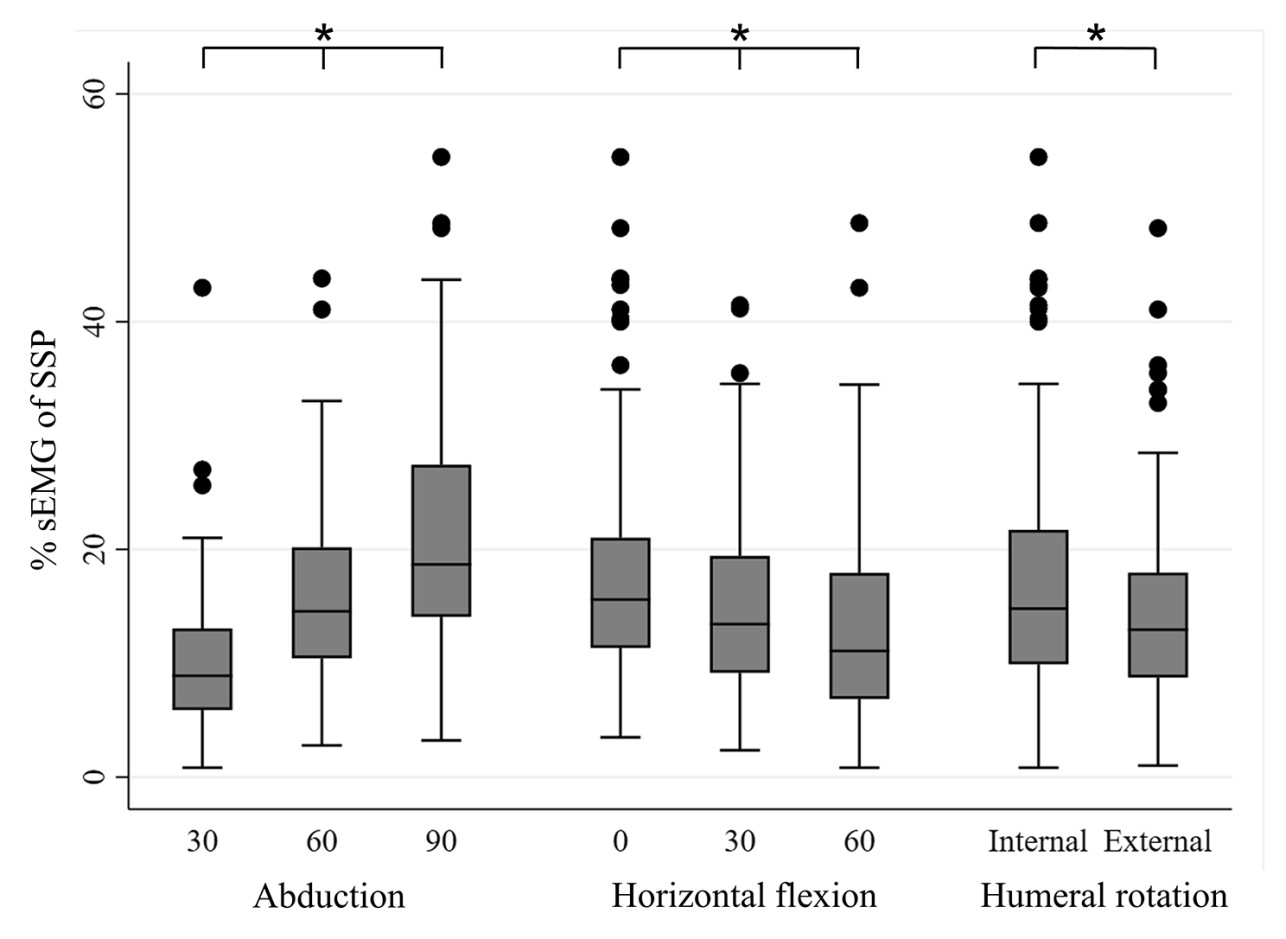
Comparison of the percentage of standardized weighted EMG (%sEMG) of supraspinatus between the groups graded by degree of shoulder abduction, degree of shoulder horizontal flexion, and humeral rotation. Error bars indicate the interquartile range (IQR) of the median. Black dots indicate values above the upper fence (1.5*IQR). * above the lines spanning between groups indicates significant *P-*values < 0.05

**Fig. 5:**
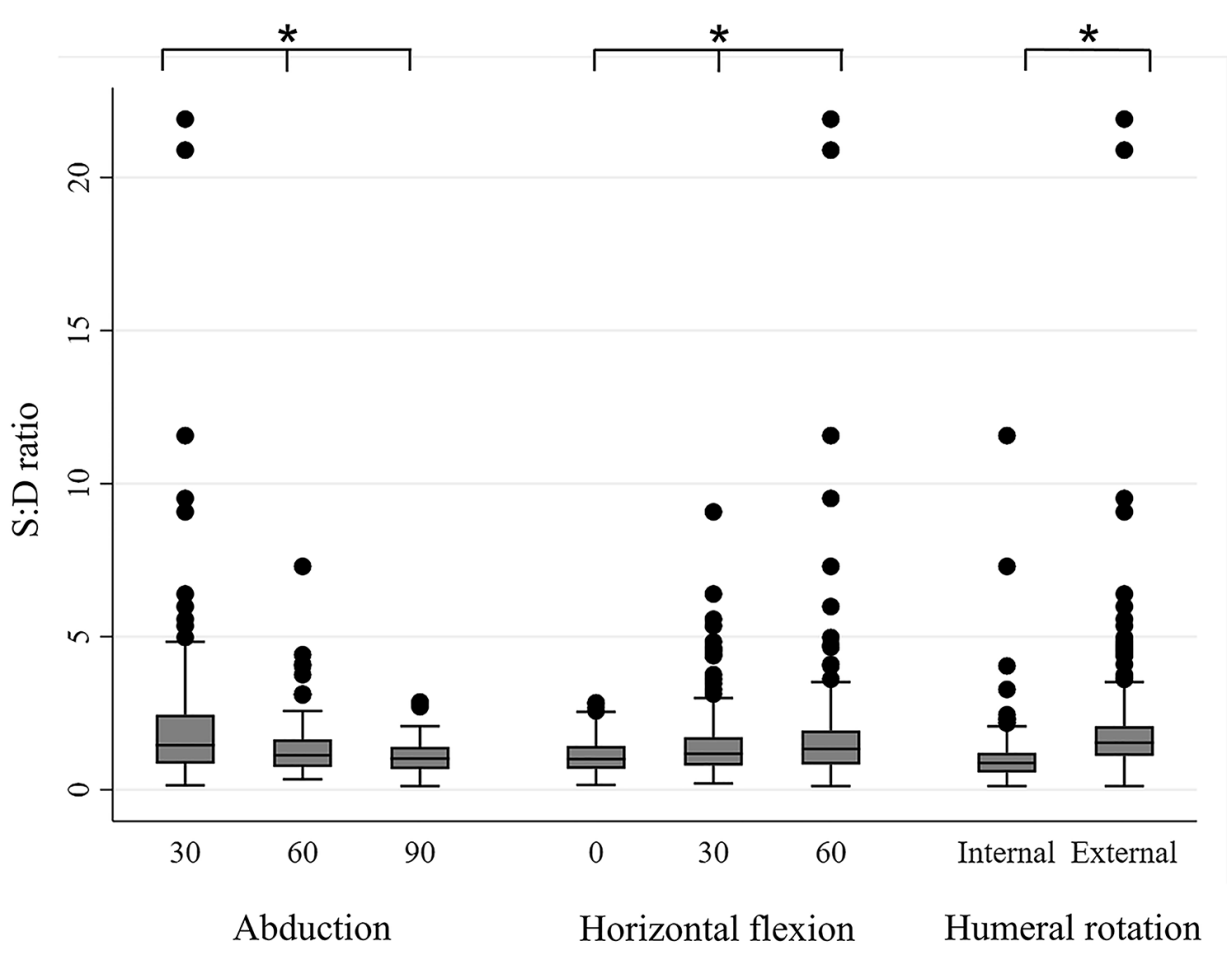
Comparison of the supraspinatus: middle deltoid (S:D) ratio between the groups graded by degree of shoulder abduction, degree of shoulder horizontal flexion, and humeral rotation. Error bars indicate the interquartile range (IQR) of the median. Black dots indicate values above the upper fence (1.5*IQR). * above the lines spanning between groups indicates significant *P-*values < 0.05

To simplify the analysis, further analysis and the S:D ratio calculation were only completed for the middle deltoid and SSP. The middle deltoid activity significantly increased in higher degrees of shoulder abduction, lower degrees of horizontal flexion, and internal humeral rotation over external rotation (*P* < 0.05; Fig. 3). SSP activity significantly increased in higher degrees of shoulder abduction, lower degrees of horizontal flexion, and external humeral rotation over internal rotation (*P* < 0.05; Fig. 4). The S:D ratio significantly increased in lower degrees of shoulder abduction, lower degrees of horizontal flexion, and external humeral rotation over internal rotation (*P* < 0.05; Fig. 5). The multilevel mixed-effects linear regression analysis revealed how the coefficients differed between groups with higher degrees of motion (60 and 90-degree abduction compared with 30-degree abduction, 30 and 60-degree horizontal flexion compared with 0-degree horizontal flexion) (Table 4).

**Table 4:** The multilevel mixed-effects linear regression revealed the differing coefficients between groups with higher degrees of motion

	Parameters	Coefficient	95% Conf. interval			P-value
**Middle deltoid**						
	**Abduction**					
	60° (compare with 30°)	7.7	6.32	–	9.08	< 0.001**
	90° (compare with 30°)	16	14.62	–	17.38	< 0.001**
	**Horizontal flexion (degrees)**					
	30° (compare with 0°)	-5.82	-7.2	–	(-4.4)	< 0.001**
	60° (compare with 0°)	-8.68	-10.05	–	(-7.3)	< 0.001**
**Supraspinatus**						
	**Abduction**					
	60° (compare with 30°)	6.08	4.87	–	7.28	< 0.001**
	90° (compare with 30°)	11.2	10	–	12.4	< 0.001**
	**Horizontal flexion**					
	30° (compare with 0°)	-2.99	-4.1	–	(-1.88)	< 0.001**
	60° (compare with 0°)	-4.84	-5.95	–	(-3.73)	< 0.001**
**S:D ratio**						
	**Abduction**					
	60° (compare with 30°)	-0.95	-1.37	–	(-0.53)	< 0.001**
	90° (compare with 30°)	-1.23	-1.65	–	(-0.81)	< 0.001**
	**Horizontal flexion**					
	30° (compare with 0°)	0.45	0.06	–	0.85	0.023*
	60° (compare with 0°)	0.86	0.47	–	1.26	< 0.001**

*Significant at level 0.05

**Significant at level 0.01

 The highest S:D ratio represented the best shoulder position to isolate the SSP from deltoid activity, and the highest ratio occurred when the shoulder was in the position of 30° shoulder abduction combined with 30° horizontal flexion and external humeral rotation, and the next highest ratio occurred at the position of 30° shoulder abduction combined with 60° horizontal flexion and external humeral rotation, which was significant with a P-value of 0.0001 from the Kruskal–Wallis test (compare among 24 shoulder positions) (Table 5). Using multiple regression with the parsimonious model, we found that factors inversely associated with the outcomes were shoulder position, body weight, and scapular plane, with P-value from F-test < 0.0001, which indicated overall significant in the regression analysis (Table 6), this information highlighted the significance of shoulder position related to S:D ratio values as proposed in our primary objective

**Table 5:** The supraspinatus: middle deltoid (S:D) ratio for 24 shoulder positions

Position no.	Abduction (degrees)	Horizontal flexion (degrees)	Rotation	Median (range)
1	30	0	IR	0.7 (0.3–1.5)
2	30	0	ER	1.5 (0.7–2.8)
3	30	30	IR	1.0 (0.3–3.3)
4	30	30	ER	3.4 (0.5–9.1)
5	30	60	IR	1.5 (0.1–11.6)
6	30	60	ER	3.0 (0.6–21.9)
7	30	SP	IR	0.9 (0.4–1.7)
8	30	SP	ER	2.5 (0.9–8.0)
9	60	0	IR	0.7 (0.4–1.3)
10	60	0	ER	1.4 (0.5–2.6)
11	60	30	IR	1.0 (0.5–1.7)
12	60	30	ER	1.6 (0.7–4.4)
13	60	60	IR	1.2 (0.3–7.3)
14	60	60	ER	1.5 (0.3–4.1)
15	60	SP	IR	0.9 (0.4–1.8)
16	60	SP	ER	1.6 (0.5–4.0)
17	90	0	IR	0.7 (1.6–1.2)
18	90	0	ER	1.4 (0.3–2.8)
19	90	30	IR	0.8 (0.2–1.2)
20	90	30	ER	1.4 (0.2–2.9)
21	90	60	IR	1.0 (0.1–1.5)
22	90	60	ER	1.2 (0.1–2.1)
23	90	SP	IR	0.9 (0.2–1.3)
24	90	SP	ER	1.4 (0.2–3.2)

P-value from Kruskal–Wallis (compared among 24 shoulder positions) = 0.0001*

*IR = internal rotation, ER = external rotation, SP = scapular plane

**Table 6:** The multiple regression with parsimonious model demonstrated variable factors associated with supraspinatus: middle deltoid (S:D) ratio

Variables	Coefficient	95% confidence interval	Standard error	P-value
**Shoulder position**	-0.05	-0.7, -0.03	0.11	< 0.001*
**Body weight (kg)**	-0.02	-0.3, -0.00	0.01	0.020*
**Scapular plane (degrees)**	-0.04	-0.8, -0.00	0.02	0.042*
**Constant**	4.6	3.15, 6.04	0.74	< 0.001*
**Adjusted R-square**	0.0545			
**P-value from the F-test**	< 0.0001*			

*Significant at level < 0.01

## Discussion

Rotator cuff tears, especially SSP tears, are a highly common cause of chronic shoulder disability, leading to decreased quality of life, decreased functionality, and increased utilization of healthcare resources [28]. Due to clinical readiness accessibility, SSP muscle strength tests are typically the first mandatory screening tool used for patients suspected of having an SSP tear [29]; the tests provide additional information and identify the need for further investigation [30]. This study aimed to determine which shoulder position can best isolate SSP from deltoid activity based on the EMG activity analysis of the periscapular musculature during resisted abduction strength testing.

To determine the best SSP muscle strength test position, the proper shoulder position must be accountable for maximizing the abducting contribution from SSP. Our result showed that both the SSP and the deltoid have increased activity related to higher degrees of abduction, but in a different magnitude, the deltoid seems to become increasingly dominant when compared to the SSP. However, resisted abduction strength still depends on both the SSP and deltoid activity. With the aim of our study to isolate SSP activity, the lower degrees of abduction should be considered to minimize the deltoid activity related to SSP activity.

Our results showed that the shoulder position of 30° shoulder abduction combined with 30° horizontal flexion and external humeral rotation best isolates the abducting activity of the SSP from the abducting activity of the deltoid. Meanwhile, the 90° shoulder abduction combined with 30° horizontal flexion and internal humeral rotation (i.e., the EC test position) manifests the least abducting activation of the SSP relative to the deltoid in proposition to the nearly lowest S:D ratio. By using S:D ratio comparison, our proposed position contributes up to 4.25-fold better in SSP isolation than the classic EC position. The clinical application of using this shoulder position in SSP strength tests can potentially improve the accuracy of physical examination using SSP testing. Nevertheless, in our analysis, we only considered the EMG activity of the middle deltoid to represent a deltoid abduction activity; we did not include anterior deltoid activity. Comparatively, a previous study suggested that the anterior deltoid contributed force only 2% for significant abduction touque [31]. Despite this difference, the shoulder position of 30° shoulder abduction combined with 30° horizontal flexion and external humeral rotation still offers the best position when considering the S:D ratio calculation with the mean deltoid activity from the anterior and middle deltoid (Supplementary Fig. 3).

The results of the present study are consistent with several published EMG studies. Wickham et al. measured EMG activity in a wide variety of periscapular muscles, demonstrating a comparable trend of muscle activation to that in our study during the shoulder abduction moment. Corresponding to the Wickham et al. study, the abduction motion also significantly increased both SSP and deltoid activity, but the magnitude of change was higher in the deltoid than in the SSP [32]. In addition, Chalmer et al. performed a specific shoulder examination test called the champagne toast test, which involves a 30° shoulder abduction combined with relative external humeral rotation. The researchers suggested that resisted abduction strength testing in lower degrees of shoulder abduction could deactivate deltoid function and isolated SSP activity [17]. Moreover, Kelly et al. conducted an EMG study of the periscapular muscles at various shoulder positions. The result of the study demonstrated better isolation of SSP activity in external humeral rotation than in internal humeral rotation [8]. Additionally, Malanga et al. examined EMG testing of the SSP and deltoid at both the empty can and Blackburn positions. The result of that examination showed that neither testing position can isolate SSP from deltoid activity [9]. Overall, a comparison of the findings suggests that our proposed position (i.e., shoulder position of 30° shoulder abduction combined with 30° horizontal flexion and external humeral rotation) better isolates the SSP than the classic EC position.

Our study revealed that the most specific shoulder position testing for isolated SSP activity is 30° shoulder abduction combined with 30° horizontal flexion and external humeral rotation. To apply these significant findings to clinical management, the SSP strength test in this preferred shoulder position would provide more specific results and the needs of further investigation, such as plain shoulder radiograph, and MRI, to diagnostic urgency conditions, such as SSP tear.

The present study has several strengths. The first is due to the limitation of previous studies when restricted planes of shoulder motion were evaluated. Our study design allows for a simultaneous comprehensive three-plane motion assessment, especially with the horizontal flexion and scapular plane, which potentially helps identify the best position of the shoulder to maximize the abducting contribution of SSP and minimize the deltoid abducting activity. Our study also advocates for each participant to serve as an internal control, and measurements were normalized for each participant. Further, the electromyography measurements were completed by a single well-trained physiotherapist, and an adequate sample size was obtained to determine the clinically significant result.

Our study also has several limitations. First, to reduce participants’ discomfort, surface-adhesive electrodes were used for EMG in our study due to their non-invasiveness. Previous studies demonstrated some relationships for estimating the EMG muscle activity between the surface and fine-needle electrode [23, 33]. Despite this variation, our significant result remains consistent with multiple previous studies that used fine-needle electrodes. Second, we did not include subscapularis in our study, which might interfere with the abduction torque in some positions [31]. Third, due to the limitations of the study design, the sample only included normal healthy individuals. However, we did not perform any further investigation to quantify that these participants are exclusively normal, though all participants were otherwise of a young age, free of shoulder pain, and in a total good health. Finally, since our experimental study included normal healthy participants with a specified age and gender, whether this finding will translate to clinical applications among patients with chronic shoulder pain and a suspected SSP tear remains unclear. In order to optimize the clinical translation of our study, we conducted both isometric testing and maximum voluntary contraction against a standardized load with accordance results. Nevertheless, further clinical testing in terms of diagnostic accuracy is still required.

### Conclusion

Application of the SSP strength test in the shoulder position of 30° shoulder abduction combined with 30° horizontal flexion and external humeral rotation is the best position to isolate the abducting activity of the SSP from the abducting activity of the deltoid. This finding might potentially help physicians seeking to diagnose patients with chronic shoulder pain and a suspected SSP tear condition.

## Electronic supplementary material

Below is the link to the electronic supplementary material.

Supplementary Material 1

## Data Availability

The datasets generated and/or analysed during the current study are not publicly available due to limitations of ethical approval involving the patient data and anonymity but are available from the corresponding author on reasonable request.

## List of Abbreviations

SSP: supraspinatus
EC: empty can test
EMG: electromyographic
S:D ratio: supraspinatus to middle deltoid ratio
BMI: body mass index
MVIC: maximum voluntary isometric contraction
%sEMG: standardized weighted EMG
