# Supplementary material for: The preferable shoulder position can isolate supraspinatus activity superior to the classic empty can test: an electromyographic study
Source: BMC Musculoskelet Disord. 2023 Apr 3;24:255. doi: 10.1186/s12891-023-06372-3 (PMC10069100; doi:10.1186/s12891-023-06372-3)
Supplement: Supplementary file 1 — Supplementary Material 1 [file 12891_2023_6372_MOESM1_ESM.pdf]

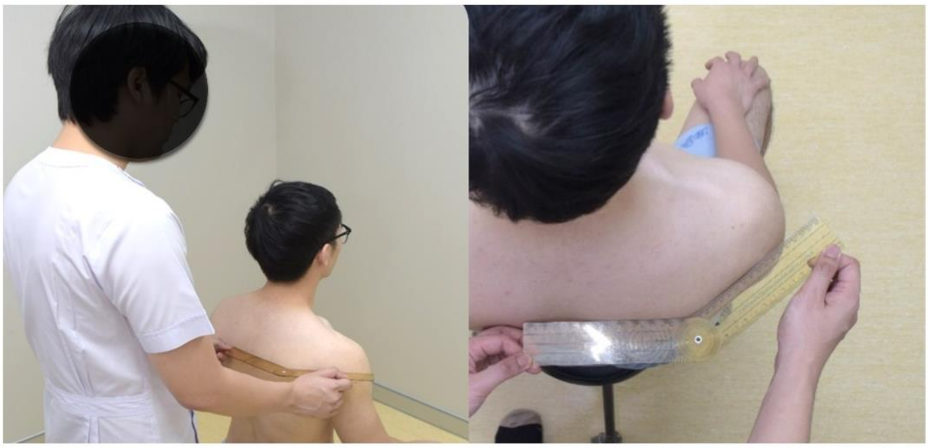

SUPPLEMENTARY FIGURE 1: Clinical photographs demonstrating the scapular plane measurement with a standard goniometer.

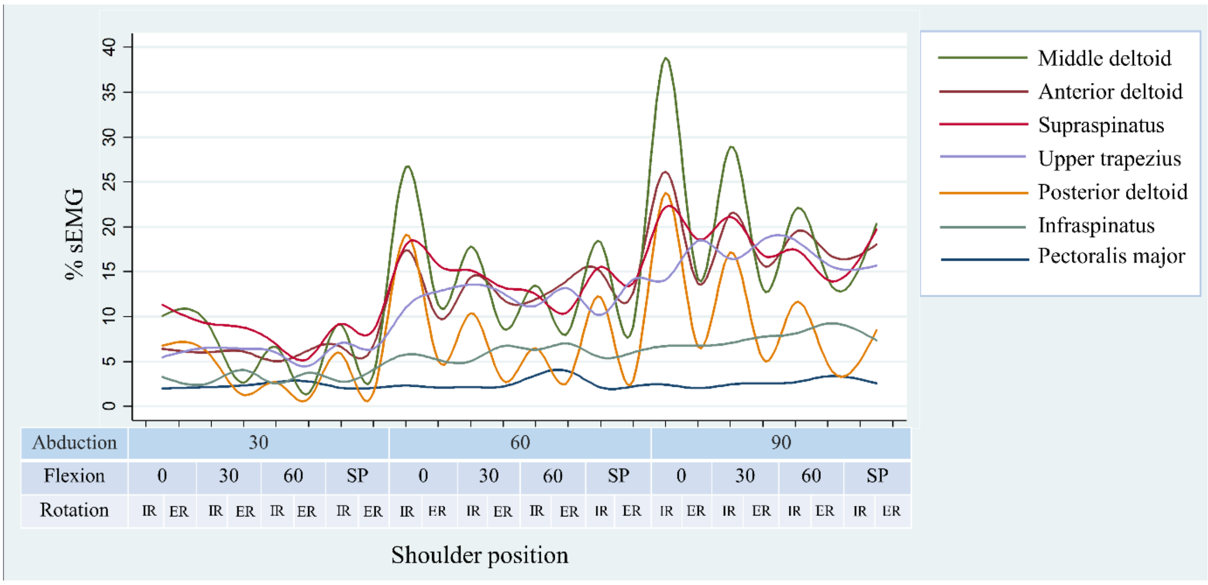

SUPPLEMENTARY FIGURE 2: The percentage of standardized weighted EMG (%sEMG) in all seven muscles (i.e., middle deltoid, anterior deltoid, supraspinatus, upper trapezius, posterior deltoid, infraspinatus, and pectoralis major) for 24 shoulder positions.

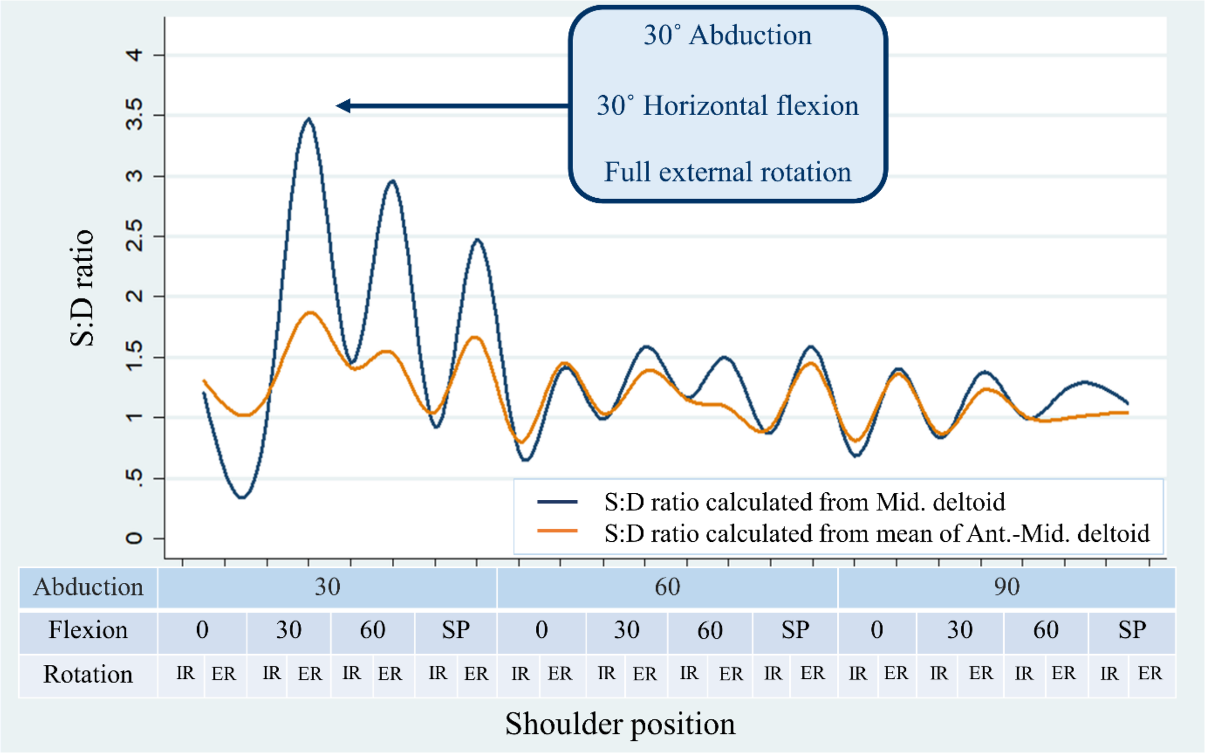

SUPPLEMENTARY FIGURE 3: The supraspinatus: deltoid (S:D) ratio for 24 shoulder positions. The shoulder position of 30° shoulder abduction combined with 30° horizontal flexion and external humeral rotation represents the highest S:D ratio calculated for the deltoid activity from either the middle deltoid or the mean of the anterior and middle deltoid.
